# Overview of Microorganisms: Bacterial Microbiome, Mycobiome, Virome Identified Using Next-Generation Sequencing, and Their Application to Ophthalmic Diseases

**DOI:** 10.3390/microorganisms13061300

**Published:** 2025-06-03

**Authors:** Kazunobu Asao, Noriyasu Hashida

**Affiliations:** 1 Department of Ophthalmology, Osaka University Graduate School of Medicine, Osaka 565-0871, Japan; kazunobu.asao@ophthal.med.osaka-u.ac.jp; 2 Department of Ophthalmology, Ikeda City Hospital, Ikeda 563-8510, Japan; 3 Department of Ophthalmology, Nakanoshima Eye Center Clinic, Osaka 569-0055, Japan

**Keywords:** ocular, microbiome, mycobiome, virome, next-generation sequencing, infectious diseases, PCR

## Abstract

This review outlines technological advances in pathogen identification and describes the development and evolution of next-generation sequencers that can be applied to the ocular microbiome. Traditional methods such as culture and PCR have limitations in detecting the full spectrum of resident microorganisms, prompting a transition toward metagenomic analysis. As microbiome research expands across body systems, the comprehensive identification of ocular bacteria, fungi, and viruses has become possible. The commensal ocular microbiome may influence disease development through changes in the immune system and ocular environment. Next-generation sequencing enables detailed microbial profiling, aiding in disease diagnosis and treatment selection. Alterations in the microbiome may also induce metabolic changes, offering insights into novel treatment methods. This review outlines the evolution of next-generation sequencing technology, summarizes current knowledge of microorganisms found on the ocular surface and in intraocular fluid, and discusses future challenges and prospects. However, the large volume of microbiome data obtained must be interpreted with caution due to possible analytical biases. Furthermore, determining whether the microbiome is truly pathogenic requires comprehensive interpretation beyond the clinical findings and results of traditional identification methods.

## 1. Introduction

The term “biome” was coined by F.E. Clements in 1916 and mentioned by F.E. Clements, V.E. Shelford, and others in 1939 [1,2]. The word “microbiome” is a combination of the terms “micro” and “biome”, and was coined by J.L. Mohr in 1952 and defined by Whipps et al. in 1988 as “a characteristic microbial community occupying a reasonably well-defined habitat which has distinct physico-chemical properties” [3,4]. The microbiome is understood to include not only the community of microorganisms but also their “theatre of activity”; this means that it includes the primary components of the microorganisms (nucleic acids, proteins, lipids, and polysaccharides) as well as their secondary metabolites (signaling molecules, toxins, and inorganic molecules) and products of changes in the surrounding environment [3,4]. Indeed, it has been suggested that the microbiome can mediate interactions between host cells and microorganisms, and that microbial metabolites play an important role in these interactions [3,4].

Humans also have their own unique biome, which is generally constantly present, although there are various individual and regional differences [5]. Human microbiome research began with research on intestinal bacteria. Since the late 1980s, culture-independent identification methods such as PCR have been developed [6]; however, owing to the limitations of primer design and differences in cycling conditions depending on the target microorganisms, PCR also has limitations regarding detection [7].

In 1987, Uhse et al. introduced the concept of targeting the 16S rRNA gene as a phylogenetic marker for new microbial community analysis [8]. The 16S rRNA gene is essential for protein synthesis in prokaryotes and contains both universally conserved as well as variable regions [8,9]. Therefore, because of its necessity for survival, size, and information content, this gene has the characteristics of an excellent phylogenetic marker [8,9]. In 1998, metagenomic analysis was introduced, which made it possible to identify microorganisms by performing PCR using primers targeting this common sequence region and sequencing the obtained amplicon. However, early analytical techniques and sequencing methods were time-consuming and expensive, and characterization of the entire gut microbiota required further advances in genome analysis methods and analytical equipment [10,11,12].

Genome sequencing began with the recognition and understanding of the helical structure of DNA [13]. In the 1970s, Frederic Sanger discovered a method to identify base pair sequences using electrophoresis based on the random incorporation of chain-terminating dideoxynucleotides by DNA polymerase during DNA replication [14]. In the 1980s, the use of Sanger sequencing, slab gel electrophoresis, and fluorescent labeling was introduced as first-generation sequencing, laying the ground for the advancement of genome analysis technology and the automation of analyses. The main disadvantage of this method was that it was time-consuming and costly to read each base one by one [15,16,17]. Nonetheless, the Sanger method is still used to confirm genome sequences because it can sequence reads longer than 500 bp, requires only small amounts of sample, and has high accuracy [18,19]. In the 1990s, second-generation sequencing, in the form of sequencing by synthesis (SBS), a method for the large-scale parallel processing of DNA sequences using short reads, was developed [17,18,19].

Since 2005, next-generation sequencers have been developed, and clonal amplification and large-scale parallel sequencing technology in flow cells has reduced run costs and improved analysis speed [20,21,22]. The second-generation HiSeq sequencing system (Illumina) has a sequencing accuracy of over 99% and is still used for clinical diagnosis [20,21,22,23,24,25,26,27]. A significant disadvantage of short-read 16S rRNA sequencing was the limited amplicon length, which made it difficult to identify microorganisms at the genus level. Long-read sequencing now allows for the analysis of the full length 16S rRNA gene amplicon, making it possible to analyze down to the species level [28,29].

In 2008, Pacific Biosciences and Oxford Nanopore Technologies developed a third-generation sequencing method that can perform single-molecule sequencing of DNA and RNA, with an average read count exceeding 1000 bp and up to hundreds of kilobases [30,31]. Third-generation nanopore sequencing identifies the waveform patterns of DNA and RNA as they pass through a nanopore, allowing real-time analysis of nucleic acid sequences [31,32,33,34]. The MinION nanopore sequencing device (Oxford Nanopore Technologies) is small, portable, and requires little sample preparation. Therefore, it can be used for analyses in clinical settings without the need for a laboratory equipped with analytical devices [34,35,36]. The sequencing accuracy (Q score) of the second-generation Illumina platform (up to approximately 99.9%) is currently higher than that of the MinION sequencer, but nanopore sequencers are now showing improvements in accuracy [33,34,35,36]. The advantage of third-generation sequencers is that they can be used in clinical settings, where rapid real-time data collection and analysis are required [33,34,35,36]. Unlike laboratory-based systems, these sequencers can be used directly in hospitals, where technicians can analyze samples on-site [31,32,33,34,35,36]. Proper sample handling and contamination prevention are critical during analysis [31,32,33,34,35,36]. Although the accuracy has improved, the accuracy of third generation sequencing is still relatively lower than that of second-generation sequencing [31,32,33,34,35,36]. Therefore, the choice of genome analysis technology and analysis equipment must be based on aspects such as the specific project requirements, number of samples reads, required accuracy, sample volume, rarity, and budget [31,32,33,34,35,36]. Additionally, biases can be introduced at each step of microbiome analysis, from sample collection to genome extraction and even during the experimental process itself, and appropriate negative controls must therefore be included [31,32,33,34,35,36].

Table 1 presents a summary of differences among the three types of pathogen detection methods.

## 2. Human Bacterial Microbiome

A wide variety of resident microorganisms reside in each part of the human body and are involved in regulating host homeostatic balance [41]. Comprehensive characterization of the resident bacterial microbiome or microbiota is considerably limited by gold standard methods such as in vitro microbial culture and PCR [7,10,11,12]. With recent technological advancements in whole genome sequencing and metagenomic analysis, which can be used to analyze several genomes in samples using next-generation sequencing, these techniques have emerged as more advantageous microbial identification methods [21,31,41]. Whole genome sequencing aims to analyze the genome of a single bacterium, while shotgun analysis and 16S rRNA metagenomic analysis are aimed at a more comprehensive identification of the microorganisms in a sample [21,31,41]. Notably, 16S rRNA metagenomic analysis can be used for identifying both bacteria and archaea, whereas shotgun analysis can also be used to identify viruses, archaea, eukaryotes, and other organisms, although it takes more time and is costlier [21,31,41].

Large-scale research began with the Human Microbiome Project (HMP) in 2007, initiated by the US NIH, followed by the MetaHIT project in 2008, which focused on characterizing intestinal bacterial flora across 13 European countries. Since then, international collaborative projects to identify the whole-body human bacterial microbiome have gained more momentum [41,42]. Consequently, it has been reported that the bacterial microbiomes that are present in each organ (skin, oral cavity, skin, conjunctiva, vagina, respiratory tract, etc.) in healthy humans could play an important role in health and immunity [5,41,43]. Differences between the bacterial microbiomes in various parts of the body have also been reported; moreover, their composition also varies depending on age, sex, and ethnicity [5,41,43,44]. These differences may be attributable to genetic predisposition as well as environmental factors such as climate, geographical conditions, diet, exercise, and medication history [43,44]. Changes are particularly noticeable when antibiotics are taken orally, and there are reports that this reduces the diversity of the intestinal bacterial flora and favors pathogens that cause opportunistic infections [44,45]. In fact, dysbiosis—defined as a breakdown of the existing microbiome—has been reported to be involved in the development of several systemic diseases such as diabetes, metabolic syndrome, inflammatory bowel disease (Crohn’s disease, ulcerative colitis), cardiovascular disease, autoimmune diseases (rheumatoid arthritis, systemic lupus erythematosus (SLE), multiple sclerosis (MS)), and neurological diseases (autism spectrum disorder, dementia, Parkinson’s disease), as well as obesity [46,47,48,49].

In the context of dysbiosis and disease, an increase in the levels of toxins derived from bacterial species and of secondary metabolites such as short-chain fatty acids and butyric acid may affect cytokine cascades and other pathways in the host environment to disrupt homeostasis [50,51,52]. In particular, short-chain fatty acids such as butyric acid, acetic acid, and propionic acid are known to have various physiological effects and affect immune defense mechanisms [52,53]. Butyric acid has been reported to promote the differentiation of regulatory T cells involved in immune control through the intestinal mucosa [54,55], while acetic acid has been reported to act on *Escherichia coli* to control immunoglobulin production and affect host defenses [56,57]. Propionic acid has antibacterial effects and promotes the activation of regulatory T cells [56,57]. Notably, the above-mentioned short-chain fatty acids have been useful in the treatment of MS [56,57].

In the context of cancer, it has been reported that changes in the abundances of various intestinal bacteria such as *Fusobacterium nucleatum*, *Streptococcus gallolyticus*, enterotoxigenic *Bacteroides fragilis*, *E. coli*, and *Enterococcus faecalis* may induce environmental mutations and even be involved in the onset of colon cancer [58,59,60,61]. Gut bacterial flora changes may affect tumor immune responses in hepatocellular carcinoma, modify immunoglobulin phenotype determination, and promote carcinogenesis [62,63,64]. Therefore, it is possible that novel alternatives to existing treatments, such as stabilizing gut bacterial flora balance, probiotic treatments for eliminating harmful metabolites, and direct introduction of gut microorganisms through fecal transplantation, may lead to a paradigm shift in the treatment of these diseases [65,66,67].

As with other parts of the body, gut bacterial flora changes have also been found to be associated with ocular diseases (chalazion, uveitis, macular degeneration, dry eye, etc.), and several studies on this topic are currently ongoing [68,69,70,71,72].

## 3. Human Mycobiome

Like bacteria and viruses, fungi coexist in various host environments and play different roles in immune support, homeostasis, and the production and decomposition of different metabolites [73,74,75]. As with bacteria, fungi can also be identified using in vitro culture and PCR, although this generally proves to be more difficult [76,77]. Very specific culture conditions are required depending on the fungus type, and even when the required conditions are met, the process generally takes between 1–2 weeks [76,77] and the positive rate in culture is low (at approximately 30–50%). PCR can confirm the presence of fungi by targeting 18S and 28S rRNA, but the identification and quantification of fungal species require specific primer sets, and there are cases where it is difficult to confirm a fungal infection, even in combination with clinical findings [76,77]. When fungal screening tests are negative, real-time PCR may not be performed; additionally, the amount of fungal DNA may be too low for PCR amplification, which could also lead to a negative result [76,77,78,79]. Notably, some studies have suggested the usefulness of β-D-glucan testing as a screening method for detecting fungi [76,77,78,79].

In the early 1990s, the targeting and sequencing of the highly variable fungal internal transcribed spacer (ITS) rRNA sequence located between the 18S, 5.8S, and 28S rRNA genes was developed as a method to comprehensively identify fungi [80,81,82,83]. With further advancements in identification techniques and equipment, it has become possible to identify fungi at the species level by deep sequencing of the ITS region using next-generation sequencers [84,85]. ITS sequencing also provides more accurate information on fungal phylotypes than 18S rRNA amplicon sequencing, making it suitable for diversity analysis [86]. However, the fungal genome comparison database is still not as comprehensive as that for bacteria, and further information will need to be shared and updated through future large-scale studies [84,85,86].

Analysis using ITS sequences has been used to identify fungi in the intestine, on the skin, and at other body other sites, as well as in various systemic, respiratory, circulatory, and pediatric, psychiatric, obstetric, and gynecological conditions [87,88,89,90]. Although bacteria and fungi coexist in the microbiome, the abundance and diversity of fungal flora are generally lower than those of bacteria [91]. In addition, like bacterial flora, fungal flora also exhibits racial, regional, and age-related differences [91]. It has also been suggested that mutations in intestinal fungal species may be involved in the development of diseases such as hypertension and diabetes, as well as smoking, metabolic disorders, and antibiotic abuse [92,93,94,95,96]. Intestinal fungal flora mutations in cases of malignant cancer may lead to intestinal mutations associated with metabolism, which, in turn, may trigger inflammation and the development of pancreatic ductal carcinoma and colorectal cancer [97,98,99]. Whole-body fungal flora analysis suggests that there may be two types of fungi: resident and pathogenic [100]. Resident fungi and their metabolites function as a barrier to remove invading pathogens and help maintain homeostasis in a diverse environment [87,88,89,90,91]. However, if the phenotype of resident fungi changes with changes in the host environment, they may become pathogenic [100]. For example, *Candida* is a resident fungus in the intestine, but it has been reported that an increase in its abundance may affect cell adhesion and the extracellular matrix, promoting local fungal adhesion [101,102]. *Candida* has also been reported to induce a proinflammatory phenotype in autoimmune diseases, worsening intestinal inflammation [101,102]. In addition, as an example of a symbiotic relationship with bacteria, *Candida albicans* can form a biofilm with bacteria such as *E. faecalis* [103,104], which suppresses the colony formation of normal bacteria such as *E. coli* and *Klebsiella* spp. and inhibits their growth [105,106]. Fungi have also been reported to be associated with more allergic changes than bacteria [107,108]. *Malassezia*, which is normal on human skin, has been reported to induce immunoglobulin E (IgE) production, act as an allergen, and cause inflammation when the host’s immunity is weakened in mucocutaneous diseases such as atopic dermatitis [107,108]. Owing to the significant changes in fungal floral composition observed in various diseases, it has been suggested that fungi may serve as potential biomarkers and therapeutic indicators correlated with clinical parameters.

## 4. Human Virome

Like bacteria and fungi, viruses are part of the microbiome and interact with other microorganisms and cells to affect the ecosystems to which they belong [109]. The term “virus” is said to have originated in 1898 when Martinus Beijerinck named the fluid containing an infectious agent “contagium vivum fluidum” [110]. In 1926, Thomas Milton Rivers described viruses as parasites that are transferred between hosts, saying that “viruses appear to be obligate parasites in the sense that their reproduction is dependent on living cells” [111]. However, the detailed shapes of viruses and bacteriophages were not known until the development of the electron microscope [112]. Initially, viruses such as the polio and yellow fever viruses were propagated in cultured infected cells, and the viral genome was identified using cloning techniques and Sanger sequencing [113,114]. Later, in order to improve sensitivity and specificity, nucleic acid amplification tests (PCR, real-time PCR, nuclear acid sequence-based amplification (NASBA), loop-mediated isothermal amplification (LAMP)), and other methods were developed [115,116]. LAMP and antibody-antigen complex detection-based methods (solid-phase enzyme immunoassays (EIAs) and enzyme-linked immunosorbent assay (ELISA)) have been further improved and have emerged as useful diagnostic tools [115,116]. CRISPR/Cas technology has been developed as a genome editing tool and is now used to identify gene mutations (including single-nucleotide mutations and structural mutations), treat genetic diseases and cancers, and support the development of novel treatments. Research in this area remains ongoing [116,117]. Modification of viral genomes is essential for elucidating viral pathogenesis, gene therapy, and vaccine development [116,117].

Viruses were primarily identified by homology analysis based on known viral genome sequences, but with advancements in sequencing technology and equipment throughout the 2000s, next-generation sequencing began to be used for viral genome analysis [118,119]. The advantage of next-generation sequencers is that they can comprehensively sequence viral DNA and RNA directly from an environmental sample, such as a sample of seawater, soil, or animal/plant tissue, without the need for cultivation. Several metagenomic and metatranscriptomic studies are currently being conducted [118,119,120]. Shotgun sequencing has been used to characterize the human gut virome, and it has been reported that viruses exist in cooperation with other microbes and the host [118,121,122]. The human virome includes bacteriophages, human viruses, as well as other RNA and DNA viruses that are highly diverse in terms of features such as genome size and virus particle structure and are also closely related to host immunity [123]. In healthy humans, viruses infect most tissues of the body asymptomatically and in a tissue-specific manner (representative examples of such viruses include human herpesviruses (HHV-6, HHV-7, and Epstein–Barr virus (EBV)), herpes simplex virus (HSV), cytomegalovirus (CMV), and human papillomavirus (HPV)) [124,125,126,127,128,129,130]. Viral infections can cause various symptoms depending on the host’s immune status, and in immunocompromised hosts, such as those with AIDS or on anticancer drugs, they generally need to be maintained in a state of remission by suppressing their proliferation below the threshold for disease onset using antiviral drugs [124,125,126,127,128,129,130]. Changes in the human virome have been suggested to be associated with many diseases, including inflammatory bowel disease, autoimmune diseases, cancer, immunodeficiency, liver disease, and skin diseases [124,125,126,127,128,129,130]. However, there are many viruses that only cause chronic infection and whose association with disease is unclear (such as the Torque Teno virus (TTV)); these viruses may become biomarkers for diseases in the future [131,132]. As analyses of the virome may also identify sequences derived from host cells, it is necessary to develop programs to identify these sequences and to set genome thresholds [133,134,135]. In addition, low genomic data quality can cause biases such as sequence fragmentation, low homology to known genes, and gene estimation errors; therefore, the appropriate selection of analysis methods is critical [136].

## 5. Clinical Studies of the Ocular Microbiome

### 5.1. Bacterial Microbiome

**Figure 1:**
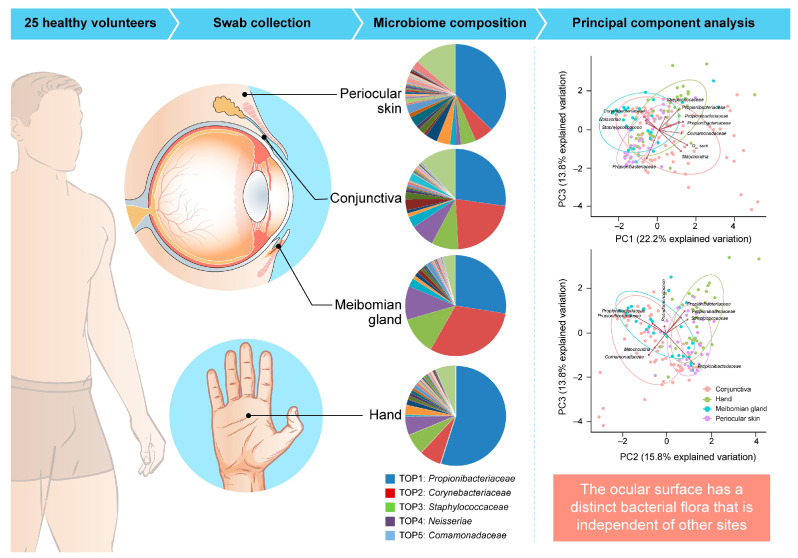
Comparison of the commensal bacterial microbiome across four body sites [137]. Samples were collected from the conjunctiva, meibomian gland, periocular area, and hand using a sterile cotton swab. Metagenomic analysis showed the relative proportions of the bacterial flora at each site and compositional differences, shown in the pie chart. The top five microorganisms composing the ocular surface bacterial microbiome at the family level were *Propionibacteriaceae*, *Corynebacteriaceae*, *Staphylococcaceae*, *Neisseriae*, and *Comamonadaceae*. Principal component analysis (PCA) was performed to assess microbial similarity among the four sites. The PCA plot shows relatively small distances between the conjunctiva (red) and meibomian gland (blue), and between the periocular skin (purple) and hand (green). In contrast, a large distance was observed between the conjunctiva and hand. The ocular surface has a distinct bacteria flora that is independent of other sites.

In our study, the commensal ocular bacterial microbiome was examined and compared across four body sites: conjunctiva, meibomian gland, periocular skin, and hand, using samples from 25 healthy volunteers [137] (Figure 1). At the family level, the predominant microorganisms on the ocular surface included *Propionibacteriaceae*, *Corynebacteriaceae*, *Staphylococcaceae*, *Neisseriae*, and *Comamonadaceae*. To investigate bacterial microbiome similarities, principal component analysis (PCA) was performed. The PCA plot revealed relatively short distances between conjunctiva (red) and meibomian gland (blue), and between periocular skin (purple) and hand (green). A larger distance was observed between the conjunctiva and hand. This study confirmed the presence of an independent and unique ocular surface bacterial flora compared to other sites (Figure 1). As previously reported, this commensal bacterial flora may change depending on the immune status of the host, and the identification of bacterial dysbiosis may be helpful for disease onset [137].

#### 5.1.1. Ocular Surface

**Figure 2:**
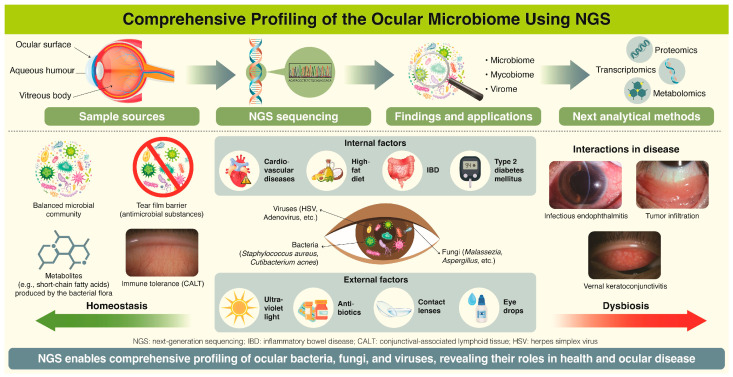
Workflow of comprehensive microbiome identification. After taking samples from the ocular surface, aqueous humor, and vitreous body, whole genomes are extracted, and the commensal bacterial flora is identified using a next-generation sequencer [137,144,145,146]. Bacterial flora resides on the ocular surface and plays a role in maintaining homeostasis [137,139,140,141]. The bacterial flora produces short-chain fatty acids such as butyric acid, acetic acid, and propionic acid, and these secondary metabolites also contribute to stabilizing the ocular surface environment [147,148]. The ocular bacterial flora is affected by both internal factors such as cardiovascular disease, immune disease, and intestinal disease, and external factors such as ultraviolet light, topical or oral antibiotics, and contact lens wear [141,142,143]. Disruption of this homeostasis may lead to infectious, allergic, or neoplastic diseases [137,144,145,146]. When investigating the relationship between changes in the microbiome and the onset of disease, multi-omics analysis combining transcriptomics, proteomics, and metabolomics may be useful for identifying biomarkers [118,119,120,149].

In the 1960s and 1970s, the positivity rate of conjunctival bacteria in in vitro culture was not high, and culturing itself was difficult [138]. With improvements in culture techniques, it became possible to culture *Cutibacterium acnes*, *Staphylococcus epidermidis*, and other bacteria from healthy conjunctival sacs, but fungi remained difficult to culture [138]. Since then, the development of PCR technology has dramatically improved identification methods [139,140]. Around 2010, reports on the characterization of the ocular surface microbiome using next-generation sequencers began to be published, and it was reported that the resident bacterial flora act as a barrier to maintain homeostasis [139,140,141]. Although the resident ocular surface bacterial flora can undergo temporary changes in response to changes in the external environment, homeostasis is maintained [141,142,143] (Figure 2).

To maintain homeostasis, the conjunctival bacterial flora depends on both intrinsic and extrinsic factors [150]. External factors that affect the ocular surface include temperature, ultraviolet light exposure, and oxidative stress and physical factors including the direct invasion of foreign bodies [143]. The ocular surface is protected by a physical barrier in the form of the tear film, innate immunity via Toll-like receptors (TLRs), antibacterial substances such as IgA, lysozyme, and lactoferrin derived from lacrimal glands and germ cells, and host defense cells such as neutrophils and macrophages in the conjunctival-associated lymphoid tissue (CALT) [150,151,152]. When the eye’s defense mechanisms fail, conjunctivitis can occur due to infectious or non-infectious causes [153]. Bacteria linked to conjunctivitis include *Haemophilus influenza*, *Streptococcus pneumoniae*, *Staphylococcus aureus*, *Neisseria gonorrhoeae*, *Moraxella* spp., *Pseudomonas aeruginosa*, *Corynebacterium* spp., and *Chlamydia trachomatis* [153]. Representative pathogens of keratitis include *S. aureus*, coagulase-negative staphylococci, *S. pneumoniae*, *P. aeruginosa*, *Moraxella* spp., *Serratia* spp., *C. acnes*, and *Mycobacterium chelonae* [154]. Cat scratch disease is an infectious disease caused by *Bartonella henselae*. It is transmitted from the host cat to humans through wounds [155]. In acute dacryocystitis, frequent pathogens include *S. pneumoniae*, *Streptococcus* spp., *H. influenzae*, and *Corynebacterium* spp. [156]. Orbital cellulitis is most commonly associated with *S. aureus*, *S. pneumoniae*, and *H. influenzae*, often involving mixed aerobic and anaerobic infections [157].

Since around 2015, advances in genome sequence platform and analytical techniques have made microbiome research more accessible and cost-effective, driving rapid growth in the field [158]. Microbial community structure varies significantly between sites, with the skin having the highest abundance and the conjunctiva having the lowest abundance. No significant differences were found between the limbus and fornix bacterial communities [158]. This review also presents variations in bacterial flora based on age, geography, publication, sampling methods, and anatomical site, and defines the core microbiome of healthy adults from published control cohort studies [158]. The conjunctival bacterial flora is reportedly less diverse than that on the body surface. As a specific core biome, the phyla Proteobacteria, Firmicutes, and Actinomyces are highly prevalent, but there are also reports of the presence of Bacteroidetes [159,160,161,162,163,164]. At the genus level, it has been reported that the genera *Corynebacterium* and *Propionibacterium* are particularly prevalent [165]. Although the reported prevalence rates vary, *Staphylococcus*, *Acinetobacter*, *Pseudomonas*, and *Streptococcus* have also been identified as being part of the core biome [151,152,153,154,155,156,157,158,159,160,161,162]. It has been reported that the breakdown of this ocular surface core bacterial microbiome could increase the risk of developing eye diseases and inflammation [162,163]. Specifically, changes in the ocular surface bacterial microbiome have been reported in infectious diseases (keratoconjunctivitis, blepharitis, trachoma, corneal ulcer, ocular trauma), corneal-related diseases (pterygium, corneal dystrophy, Fuchs endothelial dystrophy), autoimmune diseases (dry eye, Sjögren’s syndrome, Stevens–Johnson syndrome), lacrimal duct diseases (meibomian gland infarction, nasolacrimal duct obstruction), and after contact lens wear and orthokeratology treatment [161,162,163,164,165,166,167,168,169,170,171]. Dysbiosis of the conjunctival bacterial microbiome has been reported in cases of benign neoplastic tumors [137]. In patients with conjunctival MALT lymphoma, significantly higher levels of *Delftia* and fewer *Bacteroides* and *Clostridium* species compared to healthy controls [137]. Inflammatory mechanisms, including dysregulation of the NF-κB pathway and elevated IL-1β and IL-20, may contribute to the pathogenesis of conjunctival MALT lymphoma, potentially driven by microbial imbalance at the conjunctiva [137,172]. Additionally, systemic antibiotic use and abuse of multiple drugs are reported to have a stronger effect on the bacterial microbiota than local antibiotic use [173,174]. Antibiotic use may promote infection by temporarily increasing the proliferation of bacterial species that are suppressed by resident bacteria, exacerbating cytotoxicity through biofilm formation and quorum sensing, and causing environmental changes due to increased levels of certain metabolic products [175]. Disturbances in the gut bacterial microbiota led to altered production of metabolites, such as short-chain fatty acids. Past reports showed that short-chain fatty acids in the eye play an important role in regulating corneal inflammatory responses [147,148]. In addition, short-chain fatty acids have been associated with increased retinal inflammation, oxidative stress, and vascular dysfunction [147,148]. As a result, reduced levels of short-chain fatty acids could be associated with the progression of diabetic retinopathy and age-related macular degeneration [147,148]. In recent years, probiotic use has been considered for correcting dysbiosis in the development of new treatments for ocular surface diseases, and the recovery of resident bacterial flora associated with treatment may be useful not only from the perspective of therapeutic intervention but also for preventing ocular surface diseases [176,177]. Probiotics have been reported to increase secretory IgA concentrations at human mucosal sites, inhibit biofilm formation, and activate TLR signaling and are believed to stabilize the host immune response [69,178,179]. In the future, it will be important to analyze the metabolites produced during ocular surface bacterial dysbiosis, such as short-chain fatty acids and butyric acid, as by using the obtained samples for omics and proteomic analyses, it may be possible to identify further novel therapeutic targets [149].

#### 5.1.2. Aqueous Humor

Although there have been reports of bacterial flora in intraocular fluids (aqueous humor and vitreous humor) using next-generation sequencing, methods for proper sample collection, appropriate data analysis, and exclusion of bias have not yet been established and standardized. Unlike the ocular surface, the intraocular space is isolated from other parts of the body and has been considered sterile [161]. Embryologically, the retina is located at the posterior part of the eyeball and originates from part of the central nervous system [180]. Barrier mechanisms exist in both the brain and the retina to maintain a unique environment for neurotransmission (consisting of the blood–aqueous humor barrier (BAB) and blood–retinal barrier (BRB), and in the brain, the blood–cerebrospinal fluid barrier (B–CSF) and blood–brain barrier (BBB)) [181,182]. Owing to these barrier mechanisms, immune specificity has been reported in the eye that does not produce an inflammatory immune response to antigen invasion [181,182]. It has been suggested that bacteria are not present in areas protected by the blood–tissue barrier [180,181,182]. Currently, research into the presence of bacteria in the eye is being conducted, but there are several issues, such as biases inherent in next-generation sequencing itself, contamination during the sample collection and extraction process, PCR- and sequence-related biases, and the maintenance of appropriate bacterial information databases, that make it difficult to determine the presence of bacteria [183].

Collection of intraocular fluid can be done using an aqueous humor pipette or by surgical collection of aqueous humor or vitreous fluid [184,185]. Inflammatory diseases affecting the anterior chamber and vitreous, such as infections and uveitis, are key targets for analysis [186]. Recent studies have identified associations between gut bacterial microbiota and uveitis, including acute anterior uveitis, HLA B27-associated uveitis, and uveitis linked to systemic diseases such as ankylosing spondylitis, psoriatic arthritis, and juvenile rheumatoid arthritis [187,188,189,190]. Previous reports have shown that intestinal dysbiosis can cause immune signaling changes that lead to effects such as a decrease in regulatory T cell number, activation of autoreactive T cells, and immune cascade overactivity, which can in turn lead to loss of ocular immune privilege and contribute to a local inflammatory cascade, ultimately resulting in inflammation in the eye [191,192,193].

Analyses of aqueous humor samples collected during cataract surgery using next-generation sequencing have been reported [194,195,196]. Some reports suggest the presence of bacteria in the aqueous humor, while others report it being equivalent to the negative controls, and there is no complete consensus [194,195,196]. Regarding the aqueous humor bacterial microbiome, reported bacterial phyla include Acinetobacteria, Proteobacteria, Firmicutes, Acidobacteria, and Bacteroidetes. However, in some cases, samples appeared sterile or resembled the bacterial microbiota pattern seen in negative controls, raising questions about contamination and true microbial presence [194]. The genus *Propionibacterium* was identified in 70% of cataract surgery samples [195]. There have also been reports of changes in the endogenous aqueous humor bacterial flora in systemic diseases such as type 2 diabetes, although future large-scale studies are warranted [196,197].

#### 5.1.3. Vitreous Body

The vitreous is located posterior to the anterior aqueous humor. Like the aqueous humor, the vitreous body is considered sterile [198,199]; therefore, vitreous analysis requires the establishment of clean sample collection procedures that do not cause contamination during collection [200]. As vitreous surgery has progressed, sample collection techniques have improved, and reports of bacteria in vitreous samples have gradually emerged [201]. In clinical practice, intraocular inflammation is classified as infectious or non-infectious, although it can be difficult to differentiate between the two [202,203]. As antibiotic treatment is ineffective in cases of non-infectious inflammation, steroid treatment must be initiated promptly, which in turn means that an accurate diagnosis is essential [144].

Endophthalmitis is a typical example of an intravitreous infection. It is a rare condition, but prognosis is poor, and treatment must be initiated promptly [204]. Previous reports have shown that its causes are diverse, and that it can occur following cataract surgery, vitreous surgery, intraocular injections, trauma, central venous catheterization, and systemic infectious diseases (sepsis, abscess formation, urinary tract infections, etc.) [205,206,207]. Of the causative bacteria, 90% are gram-positive (70% of which are coagulase-negative staphylococci (CNS), followed by *S. aureus*, streptococci, and enterococci), and the rest are gram-negative bacilli, mainly *P. aeruginosa* [202,208,209]. The positive rate of culture is reported to be 30–40%, and that of PCR is 50–70% [202,208,209]. The severity of infection and visual outcome are related to the causative bacterial load and the toxicity of the causative bacteria; therefore, rapid identification of the causative bacteria and the presence or absence of drug resistance determine the treatment plan [203,210,211]. Despite the development of antibiotics, issues such as inflammation suppression and drug resistance still remain [203,210,211]. Endophthalmitis is treated with intravitreal antibiotic injection or vitreous surgery with antibiotic-containing irrigation solution [203,210,211]. However, there are cases of poor visual prognosis in endophthalmitis, and the development of new treatment methods is desirable. The method of administering antibiotics into the eye is based on intraocular penetration and the prevention of drug toxicity [212]. To maintain adequate bioavailability of antibiotics in cases of endophthalmitis, frequent and high-dose administration is required, which may cause side effects [212]. Recently, carbon nanomaterials have gained attention for their effectiveness against drug-resistant bacterial infections and favorable drug transport and sustained-release properties [212,213]. These materials also exhibit anti-inflammatory effects, offering a novel treatment platform for endophthalmitis [213]. In animal models, intravitreal injection of carbon nanomaterials significantly reduced microbial growth and ocular inflammation, demonstrating high biocompatibility and potential for long-acting treatment [213]. However, standardization, manufacturing costs, and environmental impact must be addressed before clinical application can advance [213]. Identification of causative bacteria using second-generation next-generation sequencing has been reported to be successful in 60–90% of cases of endophthalmitis [201,214,215]. Regarding the bacterial species involved, there are reports of both proliferation of a single species as well as mixed infections with proliferation of multiple bacterial species [144,216]. In contrast, the bacterial flora detected from samples in inflammatory diseases is equivalent to that from the extracted elution, and the presence of significant bacteria is not clear [144]. If bacterial endophthalmitis develops after ophthalmic surgery, the causative bacteria may enter through the wound and cause severe ocular inflammation [144]. Even if pathogenic bacteria are present in the vitreous, their pathogenicity is considered low unless their occupancy rate exceeds a threshold [144]. If a threshold occupancy rate of 25% is used, metagenomic analysis may be a useful tool for diagnosing inflammatory eye diseases such as uveitis [144]. In addition, it has been reported that next-generation sequencers can identify the causative bacteria in 70% of cases, even when culture is negative, suggesting that it may be useful for diagnosis [216].

Whole genome sequencing allows analysis of factors such as drug resistance genes, virulence factors, and phenotypes of isolated strains, which can improve our understanding of biofilm formation and drug resistance in bacterial species, thereby facilitating appropriate drug selection [217]. Second-generation next-generation sequencing can identify the causative bacteria at the genus level by analyzing short reads. It is faster and more accurate than traditional in vitro culture methods [33,36,201,214,215]. The advantage of this third-generation sequencer is that it can identify species using long read sequencing and can rapidly collect and analyze the data in real time, which could help to speed up antibiotic selection [34,35,36]. The MinION device has been used to identify the causative bacteria in endophthalmitis, successfully identifying bacteria even in culture-negative cases. This capability may support more effective treatment and suggests a transition from research use to clinical diagnostic application in hospitals [218,219,220,221,222,223]. However, the base determination accuracy of the MinION sequencer is slightly lower than that of second-generation systems, and the possibility of a high error rate remains [218,219,220,221,222,223]. Therefore, it is necessary to exclude biases such as the possibility that the bacteria load is too low due to the small amount of vitreous sample as well as the possibility of contamination during sample processing and genome extraction, and a comprehensive judgment including culture and clinical findings is required for diagnosis [224].

### 5.2. Mycobiome

In our study of 25 healthy volunteers, we investigated the commensal fungal microbiome of the conjunctiva [225]. The dominant genera identified were *Malassezia*, *Corallomycetella*, and *Byssochlamys*, with *Malassezia* consistently predominant. In the human body, *Malassezia* exists in the sebum- rich parts of the human skin and is involved in the metabolism of lipids to maintain homeostasis. Similarly, the conjunctival fungal microbiome, including conjunctiva-associated lymphoid tissue, contributes to ocular immune defense and maintains surface homeostasis. However, overgrowth of *Malassezia* may disrupt the ocular surface barrier. Fungi comprise only approximately 1% of the total ocular microbiome, which can make their identification difficult. Furthermore, current fungal databases and identification systems remain underdeveloped. Next-generation sequencing can be an effective tool for pathogen identification, but great care must be taken to eliminate bias and contamination [225].

#### 5.2.1. Ocular Surface

Although bacteria and fungi coexist in the microbiota, fungi are reported to be less abundant and diverse than bacteria at all sites [91]. Identification of ocular surface fungi is based on culture and PCR using swab and corneal scraping samples, but the positivity rate is low, at approximately 20% [226,227]. Until now, comprehensive identification of resident fungal flora has been difficult owing to differences in the required culture conditions for each fungus, low abundance, and difficulties related to primer design [226,227]. In 2019, the characterization of ocular surface fungal flora in healthy individuals was reported using next-generation sequencing, with identification down to the genus level achieved for 70% of the samples [228]. Indeed, shotgun sequencing revealed that the microbiota components were predominantly bacterial, with less than 1% being fungal or viral [153]. The genera *Aspergillus*, *Setosphaeria*, *Malassezia*, and *Haematonectria* were identified across all samples [228]. Another study reported that the genus *Malassezia* accounts for the majority of ocular surface fungi (75%), followed by *Rhodotorula*, *Davidiella*, *Aspergillus*, and *Alternaria* [229].

In a report on ocular surface diseases, next-generation sequencing identified fungi in 70% of corneal scraping samples from patients with keratomycosis [230]. A comparison with culture results showed that the identification of causative bacteria was consistent in 30% of cases, indicating that next-generation sequencing could be an option for diagnosis and treatment [230]. In addition to causative pathogenic bacteria, the presence of opportunistic pathogens such as *Emericella*, *Fusarium*, *Malassezia*, *Aspergillus*, and *Cetosphaeria* has also been reported [230]. In addition, in autoimmune diseases such as Sjögren’s syndrome and dry eye, a decrease in fungal diversity is associated with an increase in disease activity, which in turn has been reported to be correlated with disease severity [225,231].

The ocular surface serves as the first line of defense after the eyelid, directly exposed to environmental fungi and hosting diverse fungal communities [232]. The occupancy rate of ocular surface flora is reported to change with the severity of meibomian gland infarction and to be related to the progression of dry eye [233]. The genus *Malassezia* is the most common genus identified in human skin and also accounts for the majority of the ocular surface fungal flora [225,228,229,230,231,232]. *Malassezia* induces immune IgE production and is involved in maintaining the balance of inflammation at mucosal sites [107,234]. CALT, which is part of the ocular immune system, secretes secretory IgA to protect against external antigens, stabilizes the tear mucin layer, and suppresses bacterial adhesion and allergies [235].

Environmental allergens can enter cells through the corneal and conjunctival epithelium, and dendritic cells are the main effectors of the initial immune response [236]. After capturing antigens, these cells migrate to lymph nodes and present the antigens to naive CD4 T cells, initiating an immune response [237,238]. Consequently, differentiation into allergen-specific Th2 cells is induced, resulting in allergic inflammation [237,238]. Th2 and Th9 cells act on adaptive immune responses in the conjunctiva, inducing type 2 allergic immune-mediated inflammatory processes, including the secretion of cytokines such as thymic stromal lymphopoietin and interleukin (IL)-33 [239,240]. For example, in the conjunctival epithelium, the conjunctival epithelial barrier is disrupted through the IL-33/ST2/IL-9/IL-9R signaling pathway, which contributes to the exacerbation of inflammation [241]. Therefore, excessive allergic responses induce inflammation, resulting in a cascade that worsens the ocular surface environment [241]. Vernal keratoconjunctivitis is a severe form of allergic conjunctivitis characterized by proliferative changes in the conjunctiva [145]. In some cases, a shield ulcer is detected on the upper cornea, caused by the giant papillae on the upper tarsal conjunctiva [236,239]. In cases with conjunctival MALT lymphoma, the fungal dysbiosis at the conjunctiva was detected and allergic conjunctivitis was frequently observed in affected patients [145]. Similar patterns of fungal shifts, particularly involving *Malassezia*, have been implicated in the pathogenesis of atopic dermatitis and may serve as potential biomarkers [242]. *Malassezia* may also play a role in disease development on the ocular surface, and monitoring its occupancy could provide a disease biomarker that is useful for therapeutic intervention [243].

#### 5.2.2. Aqueous Humor and Vitreous Body

As mentioned above, there is still no consensus on whether microbial and fungal flora truly exists in the anterior chamber and intraocular fluids [180,181,182,183]. In this section, we will describe the findings on fungal flora without separating the aqueous and vitreous humors.

There have been some studies on fungal identification in the aqueous humor using multiplex PCR [244]. Targets include fungal 18S/28S rRNA, the *Candida* genus, *C. glabrata*, *C. krusei*, *Aspergillus*, and *Fusarium*, which are useful for diagnosing infectious diseases of unknown cause [244]. Notably, the positive identification rate of multiplex PCR is higher in the aqueous humor than in the vitreous humor, and it may therefore be possible to identify pathogens in the aqueous humor, which is easier to collect [245]. Next-generation sequencing allows comprehensive identification of bacteria and fungi by simultaneous sequencing of 16S rRNA and the ITS region, which is a major advantage [246]. The required clinical sample volume is small, with a minimum of 20 µL for the aqueous humor and 100 µL for the vitreous, making it a useful identification method that is faster than culture or PCR [244].

In relation to diseases, we will discuss fungal endophthalmitis, the most serious type of fungal infection. Fungal invasion into the eye can occur directly during surgery, although this is rare. Fungal infections often occur after fungemia develops in immunocompromised patients, such as those undergoing cancer treatment, steroid therapy, or living with conditions like liver abscess, chronic renal failure, or HIV, and can spread from distant organs to the eye [247]. The most common causative fungi are *Candida*, *Aspergillus*, and *Fusarium*, and many of these are associated with cataract and vitreous surgery [205,248,249]. Compared to bacteria, fungi develop more slowly and cause less inflammation, making them difficult to detect [205]. Fungal endophthalmitis is rare but requires urgent treatment [247]. Culture methods require specific culture conditions and settings and a long incubation period, and PCR methods require optimization of primer sets, making comprehensive fungal identification difficult [250]. As described in the section on bacterial endophthalmitis, next-generation sequencing can be used to identify causative bacteria from the aqueous humor or vitreous humor even in culture-negative cases, making it a useful option for diagnosis [214,216]. Furthermore, ITS region analysis allows for species-level analysis and identification in a short time because of the long reads [251]. In cases of fungal endophthalmitis, detection of the pathogen is essential [146]. In particular, the choice of antibiotics differs depending on whether the cause is bacterial or fungal [146]. In culture for fungi, the results are obtained approximately 1 week after specimen submission [146]. While PCR has high specificity and enables rapid identification, only specific species can be identified depending on the primer design [146]. ITS deep sequencing allows identification of the pathogen within 3 d, and rapid resolution of inflammation can be observed by switching from antibiotics to antifungals to provide an appropriate antibacterial spectrum [146]. These results support the usefulness of ITS1 sequencing in the rapid identification of pathogens in cases of fungal endophthalmitis and provide useful information for the selection of appropriate antifungals [146]. When selecting antibiotics, shotgun metagenomic analysis can also be used to analyze drug resistance-related genes, which may be useful for selecting the most appropriate therapeutic intervention [252]. Unlike bacteria, for which comprehensive databases and analytical methods are available, including information on drug resistance genes, similar platforms for fungal identification remain underdeveloped [85,252]. Additionally, shotgun metagenomic analysis is problematic in that it is costly and data analysis takes a long time [85,252]. To determine whether the fungus obtained is a pathogen, a comprehensive judgment is required based on the results of standard methods such as in vitro culture and PCR [223].

### 5.3. Virome

In our study, we analyzed samples from the ocular surface, aqueous humor, and vitreous body to investigate the ocular virome. A wide range of viral subtypes was detected, including Torque teno virus, herpesvirus, and papillomavirus. Although no formal paper has been published, our data suggest that herpesvirus shedding occurs even in healthy individuals, not only in patients with known herpes infections. This raises the potential for viral presence in tears to be used in monitoring treatment efficacy. However, many of the detected viruses may represent false positives. Eye-derived samples yield only small amounts of nucleic acid, increasing the risk of contamination during extraction and analysis. In contrast, if the virus copy number in the specimen is very high, reliable detection is possible. Therefore, further technological advances, improved next-generation sequencing capabilities, and standardized analytical methods are needed to enhance virome research.

#### 5.3.1. Ocular Surface

Viral eye diseases cover a wide range of conditions, including blepharitis, conjunctivitis, keratitis, uveitis, cataracts, and retinitis [253], and commonly reported causative viruses include HSV, varicella zoster virus (HZV), CMV, EBV, and adenovirus [254,255,256,257,258,259]. EBV is a common cause of conjunctivitis. Examples of viral conjunctivitis that can develop into epidemics include epidemic keratoconjunctivitis (caused by adenovirus types 8, 19, and 37), pharyngoconjunctival fever (caused by adenovirus types 3, 4, and 7), and acute hemorrhagic conjunctivitis (caused by enterovirus type 70 and Coxsackie A24 variants) [254,255,256,257,258,259]. In patients with HSV keratitis, the virus is shed and can be detected in tears using PCR [260]. Similarly, HCV has been detected in the tears of patients with dry eye and is reported to be associated with the disease [261]. EBV infection has been reported to be related to lacrimal gland lymphocyte proliferation in Sjögren’s syndrome and may cause aqueous-deficient dry eye [262]. Inflammation from systemic viral infections, such as influenza, dengue, chikungunya, Zika, and COVID-19, can affect not only the ocular surface but may also extend to the posterior segment, resulting in ocular disease [263].

Next-generation sequencing for identification of viral flora is different from other sequence-dependent approaches and can be applied to viruses for which sequence information is not available [253,254]. Advances in analytical technology and pipelines as well as artificial intelligence-based approaches have led to reports of associations between intestinal viral flora and autoimmune and cardiovascular diseases, but there have been fewer reports on ocular viral flora, including those on the ocular surface and in the intraocular space [253,254]. In 2016, Doan et al. reported the presence of multiple sclerosis–associated retrovirus (MSRV), human endogenous retrovirus K (HERV-K), TTV, Merkel cell polyomavirus (MCV), HPV, and Abelson murine leukemia virus [165]. In 2021, Siegal et al. identified MCV in the conjunctiva and confirmed its presence using PCR [264]; however, the possibility of these viruses being host-derived cannot be ruled out, and further investigation is required into their pathological significance [165,265].

Regarding age-related changes, a recent study reported that, unlike that of bacterial flora, the localization of viral flora on the ocular surface of the pediatric conjunctive is more scattered [266]. Although the diversity and composition of viral flora did not change with the administration of antibacterial or antiviral antibiotics, changes over time have been reported on an annual basis [266]. Like bacterial and fungal flora, conjunctival viral flora may change with age, and long-term follow-up is therefore necessary to assess homeostasis [266].

#### 5.3.2. Aqueous Humor and Vitreous Body

Identification of the causative virus in intraocular fluids is essential for the treatment of ocular inflammatory diseases and eye infections. In recent years, strip PCR has become widely used because of its simplicity [245,267]. It uses intraocular fluid to simultaneously amplify multiple target DNA regions within a single reaction tube, making it possible to simultaneously detect multiple pathogens (bacteria, fungi, and viruses) [245,267]. Currently available primer sets for viruses include those for HSV type 1 and 2, varicella zoster virus (VZV), EBV, CMV, HHV types 6, 7, and 8, human T-cell lymphotropic virus (HTLV)-1, and human adenovirus (HAdV) [245,267]. Direct strip PCR technology, which allows direct PCR without DNA extraction, has also been used to identify causative viruses in cases of uveitis and is expected to shorten the time needed for identification [268]. Multiplex PCR can also be used to detect antibiotic resistance genes simultaneously, thus providing useful information for antibiotic selection [268]. It is a relatively simple PCR methodology that takes into account reported viral genome sequences, and although copy numbers can be estimated later using quantitative PCR, it is not the ideal method for comprehensive characterization of the viral flora [245,264,267,268].

There are very few detailed reports on the use of next-generation sequencing for the identification of viral flora in intraocular fluid. It has been reported that the rubella virus causes Fuchs heterochromia, and next-generation sequencing has shown the presence of rubella virus RNA in the aqueous humor of patients, indicating a relationship with the disease [269,270]. There has also been a report on the identification of viral flora in the vitreous fluid of patients with post-fever retinitis [270]. Detected viral families include *Myoviridae*, *Siphoviridae*, *Phycodnaviridae*, *Herpesviridae*, *Poxviridae*, *Iridoviridae*, *Podoviridae*, *Retroviridae*, *Baculoviridae*, and *Flaviviridae* [271]. In this study, healthy controls were patients who underwent ophthalmic surgery for non-infectious vitreous diseases such as macular hole and rhegmatogenous detachment. Members of the genus *Lymphocryptovirus* were significantly overrepresented in post-fever retinitis, and many other viral changes were also confirmed, suggesting the possibility of changes associated with the host immune response [271]. In addition, TTV was detected in half of the culture-positive samples and all culture-negative samples in endophthalmitis [272]. It has been reported that the presence of TTV in endophthalmitis may increase the risk of retinal detachment and the need for reoperation, leading to poor prognosis [273]. TTV has also been reported to be involved in inflammation, MS, and panuveitis [272,273,274]. However, although it may be constantly present on the ocular surface, the route of its entry into the eye has not yet been clearly reported [273,274]. Like TTV, MCV has also detected in cases of postoperative endophthalmitis [275]; it has also been reported to be present in the eyelid area of patients with primary eyelid Merkel cell carcinoma and may be involved in neoplastic growth in the eye [275]. Nonetheless, further research is needed to clarify the details of their roles in intraocular inflammation.

Despite advances in virus identification methods, it remains difficult to consistently identify viral flora. There are still several limitations regarding the technical aspects and interpretation of the data obtained using next-generation sequencing [276]. There is no consensus among researchers on the analysis methods and threshold settings of the obtained information, and so it is important to eliminate bias [276]. It is also difficult to determine whether the obtained sequence corresponds to a viral genome or that of the host, which makes it essential to check the quality of the obtained data [277]. Databases such as RefSeq have been established to enable the comparison of viral sequences, and tools such as PHASTEST have been developed to search for host genome-derived prophage sequences [278]. Other tools include the Basic Local Alignment Search Tool and DIAMOND aligner for genome homology searches, as well as the Genome Aggregation Database, which uses machine learning based on base sequence frequency to detect viruses without known homology. Continued database revisions and advances in analysis methods are also expected in the future [37,38,39,40,279,280,281,282].

Table 2 presents the overview of published articles regarding the ocular microbiome and next-generation sequencing.

## 6. Future Prospects

The findings in the literature indicate that next-generation sequencing may be a useful alternative to conventional methods for identifying pathogenic organisms. The major advantage of next-generation sequencing-based analysis is that comprehensive analyses of bacteria, fungi, and viruses are possible. With advancements such as reduced time and cost, increased sample throughput, the shift from short to long reads, and improvements in portability and ease of use, the potential for the real-time identification of pathogens is increasing, potentially enabling a transition from laboratory-based to clinical applications.

However, several details still require attention. Comprehensive analyses can identify both live and dead bacteria, and it is also important to judge whether the identified bacterial flora represents resident bacteria or pathogenic bacteria. In terms of simplicity and ease-of-use, third-generation nanopore sequencers are portable and capable of real-time analysis, but contamination during sample processing may affect the results. Biases may also arise from factors such as sample quantity, collection methods, differences in protocols, and equipment variability. To minimize these effects, standardized procedures and rigorous quality control are needed. Bioinformatics analysis pipelines and machine learning algorithms are used to compare with existing databases to identify the microbiota, but while there are databases for bacteria, this is not yet the case for fungal and viral flora. It is crucial to evaluate NGS methods in terms of sensitivity, specificity, cost, and turnaround time, and to interpret results alongside traditional methods such as culture, PCR, and clinical findings. Finally, an important point regarding analysis using next-generation sequencers is that it is unclear whether many of the confirmed dysbioses are the cause or result of the onset of disease. To investigate this, multi-omics analysis, including transcriptomics, proteomics, and metabolomics, may help identify new biomarkers and metabolic pathways, potentially leading to novel treatments.

In conclusion, this review highlights the utility of next-generation sequencing in identifying ocular bacterial, fungal, and viral flora. Advances in analytical methods and equipment have enabled more accurate and prompt identification of ocular microorganisms. However, effective clinical application requires integration with traditional diagnostic methods and comprehensive evaluation of ocular microbiota. Continued accumulation of data and large-scale studies are essential to further advance this field.

## Figures and Tables

**Table 1:** Summary of differences among the three types of pathogen detection methods [13,14,15,16,17,18,19,20,21,22,23,24,25,26,27,28,29,30,31,32,33,34,35,36,37,38,39,40].

Generation	First-Generation Sequencing	Second-Generation Sequencing	Third-Generation Sequencing
Detection methods	Sanger sequencing capillary electrophoresis	Bridge PCR Sequencing by synthesis	DNA elongation in a microwell Nanopore sequencing
Since	Late 1990s	~2005	~2017
PCR	Yes	Yes	No
Read lengths	~1000 bp	100–200 bp	>10 Kbp
Characteristics	Low reading error rate and relatively long read length approach	Short read length approach Large-scale parallel sequencing Reduced run costs Improved analysis speed	Long read length approach; Fastest sequencer; Whole-genome scan within 15 min
Disadvantages	High run costs Low analysis speed	Identify microorganisms at the genus level Laboratory-based study	Relatively high error rates
Sequencing accuracy (Q score)	>Q20	>Q31	>Q21
Representative equipment	SeqStudio Genetic Analyzer (Thermo Fisher Scientific)	454 sequencing (454 Life Sciences) NovaSeq (Illumina) Ion Torrent Sequencing (Thermo Fisher)	Sequel II (Pacific Biosciences) MinION platform (Oxford Nanopore Technologies)

The sequencing quality score of a given base, Q, is defined by the following equation: Q = −10log10(e), where e is the estimated probability of the base call being wrong.

**Table 2:** Overview of published articles on ocular microbiome and next-generation sequencing.

Authors	Participants	Locations	Major Findings	References	Category	Generation
Zhou Y et al., 2014	105 healthy volunteers 115 patients with suspected trachoma	Conjunctiva	In trachomatous disease, changes in the conjunctival microbiome could occur	[140]	Bacterial Microbiome	Second
Doan T et al., 2016	107 healthy volunteers	Conjunctiva	On the healthy ocular surface, Corynebacteria, Propionibacteria, and coagulase-negative Staphylococci were the predominant organisms. TTV were also detected.	[164]	Bacterial Microbiome Virome	Second
Deng Y et al., 2021	41 patients with cataract, glaucoma and AMD	Conjunctiva Aqueous Humor Plasma Skin	*Cutibacterium acnes* was the most abundant. Complex community of bacteria might be present inside the eyes.	[195]	Bacterial Microbiome	Second
Deshmukh D et al., 2019	34 patients with endophthalmitis 30 participates with non-infectious retinal disorders as controls	Vitreous Fluid	Culture-based diagnosis was achieved in 44% of cases. NGS diagnosed the presence of microbes in 88% of cases.	[214]	Bacterial Microbiome Mycobiome	Second
Low L et al., 2022	23 patients with suspected endophthalmitis	Aqueous Humor Vitreous Fluid	At genus level, the coincidence between culture and 16S Nanopore, Nanopore WGS, and Illumina WGS were 75%, 100%, and 78%, respectively.	[217]	Bacterial Microbiome	Second and Third
Hao X et al., 2023	34 eyes with endogenous endophthalmitis	Aqueous Humor Vitreous Fluid	NTS and culture detected pathogens in 89.28% and 35.71% of cases. The average detection time of NTS (1.11 days) was shorter than that of culture (2.50 days).	[219]	Bacterial Microbiome Mycobiome	Third
Eguchi H et al., 2023	8 patients with clinically diagnosed bacterial keratoconjunctivitis	Ocular Surface Specimens	In 66% of culture-negative cases, the smear positivity closely resembled the MinION results. In 80% of culture-positive cases, culture and sequencing results were consistent.	[224]	Bacterial Microbiome	Third
Shivaji S et al., 2019	34 healthy volunteers	Conjunctiva	The genera *Aspergillus*, *Setosphaeria*, *Malassezia*, and *Haematonectria* were present.	[228]	Mycobiome	Second
Wang Y et al., 2020	90 healthy volunteers	Conjunctiva	Two phyla, Basidiomycota and Ascomycota, and five genera, *Malassezia*, *Rhodotorula*, *Davidiella*, *Aspergillus*, and *Alternaria*, were identified, accounting for >80% of the fungal microbiome.	[229]	Mycobiome	Second
Prashanthi GS et al., 2019	25 healthy controls 35 patients with fungal keratitis patients	Conjunctiva Corneal Epithelium	Alteration in the fungal microbiota was observed both at the phylum and genera levels. The ocular microbiome analysis identified 11 genera.	[230]	Mycobiome	Second
Siegal N et al., 2021	20 anophthalmic and 20 fellow-eye	Conjunctiva	TTV and MCPyV were detected frequently in healthy and anophthalmic conjunctiva.	[265]	Bacterial Microbiome Virome	Second
Doan T et al., 2020	Not available	Conjunctiva	The structure of the ocular surface virome was not altered after azithromycin treatment	[266]	Bacterial Microbiome Virome	Second
Arunasri K et al., 2020	19 healthy volunteers 9 patients with post fever retinitis	Vitreous Fluid	An increase in abundance of anti-inflammatory and antimicrobial genera and decrease in proinflammatory genera were detected compared with that in healthy controls.	[271]	Bacterial Microbiome	Second
Lee et al., 2014	21 patients with presumed infectious endophthalmitis 7 patients with noninfectious retinal disorders or culture-negative endophthalmitis	Aqueous Humor Vitreous Fluid	57.1% of culture-positive and 100% of culture-negative samples demonstrated the presence of TTV.	[272]	Bacterial Microbiome Virome	Second
Lee CS et al., 2020	50 patients with postprocedural endophthalmitis	Aqueous Humor Vitreous Fluid	In post-procedure endophthalmitis, a higher load of bacteria other than that of *S. epidermidis* and the presence of TTV DNA are associated with worse outcomes.	[273]	Bacterial Microbiome Virome	Second

Abbreviations: AMD: age-related macular degeneration, MCPyV: Merkel cell polyoma, NGS: next-generation sequencing, NTS: nanopore-targeted sequencing, TTV: torque teno virus, WGS: whole genome sequencing.

## Data Availability

All data related to this work are presented and published here.
